# Genes Encoding Transcription Factors TaDREB5 and TaNFYC-A7 Are Differentially Expressed in Leaves of Bread Wheat in Response to Drought, Dehydration and ABA

**DOI:** 10.3389/fpls.2018.01441

**Published:** 2018-09-27

**Authors:** Lyudmila Zotova, Akhylbek Kurishbayev, Satyvaldy Jatayev, Gulmira Khassanova, Askar Zhubatkanov, Dauren Serikbay, Sergey Sereda, Tatiana Sereda, Vladimir Shvidchenko, Sergiy Lopato, Colin Jenkins, Kathleen Soole, Peter Langridge, Yuri Shavrukov

**Affiliations:** ^1^Faculty of Agronomy, S.Seifullin Kazakh AgroTechnical University, Astana, Kazakhstan; ^2^Karaganda Research Institute of Plant Industry and Breeding, Karaganda, Kazakhstan; ^3^School of Agriculture, Food and Wine, University of Adelaide, Urrbrae, SA, Australia; ^4^College of Science and Engineering, Biological Sciences, Flinders University, Bedford Park, SA, Australia

**Keywords:** ABA treatment, dehydration, drought, gene expression, grain yield, qRT-PCR, stomatal conductance, wheat

## Abstract

Two groups of six spring bread wheat varieties with either high or low grain yield under the dry conditions of Central and Northern Kazakhstan were selected for analysis. Experiments were set up with the selected wheat varieties in controlled environments as follows: (1) slowly progressing drought imposed on plants in soil, (2) rapid dehydration of whole plants grown in hydroponics, (3) dehydration of detached leaves, and (4) ABA treatment of whole plants grown in hydroponics. Representatives of two different families of transcription factors (TFs), *TaDREB5* and *TaNFYC-A7*, were found to be linked to yield-under-drought using polymorphic Amplifluor-like SNP marker assays. qRT-PCR revealed differing patterns of expression of these genes in the leaves of plants subjected to the above treatments. Under drought, *TaDREB5* was significantly up-regulated in leaves of all high-yielding varieties tested and down-regulated in all low-yielding varieties, and the level of expression was independent of treatment type. In contrast, *TaNFYC-A7* expression levels showed different responses in the high- and low-yield groups of wheat varieties. *TaNFYC-A7* expression under dehydration (treatments 2 and 3) was higher than under drought (treatment 1) in all high-yielding varieties tested, while in all low-yielding varieties the opposite pattern was observed: the expression levels of this gene under drought were higher than under dehydration. Rapid dehydration of detached leaves and intact wheat plants grown in hydroponics produced similar changes in gene expression. ABA treatment of whole plants caused rapid stomatal closure and a rise in the transcript level of both genes during the first 30 min, which decreased 6 h after treatment. At this time-point, expression of *TaNFYC-A7* was again significantly up-regulated compared to untreated controls, while *TaDREB5* returned to its initial level of expression. These findings reveal significant differences in the transcriptional regulation of two drought-responsive and ABA-dependent TFs under slowly developing drought and rapid dehydration of wheat plants. The results obtained suggest that correlation between grain yield in dry conditions and *TaNFYC-A7* expression levels in the examined wheat varieties is dependent on the length of drought development and/or strength of drought; while in the case of *TaDREB5*, no such dependence is observed.

## Introduction

The two phenomena of drought and dehydration, sometimes considered synonymous, result when there is a shortfall in the amount of available soil or air moisture levels required to meet plant metabolic and transpirational demands. For the purpose of the current study, drought is defined as the relatively slow and gradual process of water withholding from the whole intact plant growing in soil or hydroponically. In contrast, dehydration refers to the removal of the whole plant from the soil or hydroponic medium, or the detachment of leaves, resulting in rapid water loss or ‘dehydration shock.’ Despite the common basic cause, drought and dehydration have the obvious difference of whether active roots are present, which are able to transport water and solutes to upper parts of the plant, and participate in signaling. By this definition, drought-affected plants can often recover after re-watering, but for dehydrated plants removed from the root medium, or dehydrated detached leaves, the stress can be fatal.

Intact wheat plants growing in soil under drought maintain the typical signaling systems that provide cross-talk between plant organs by co-ordinated changes in the expression of hundreds or thousands of genes in different plant organs (Stockinger et al., 1997; Mariaux et al., 1998; Thompson et al., 2000; Gürel et al., 2016). In contrast, rapid or ‘shock-like’ dehydration of plants or detached leaves has been used to study the reaction of plant cells with no or minimal influence of signaling systems from other parts of the plant (Gürel et al., 2016). The early sensing of shock-like gene expression responses has also been examined in *Arabidopsis thaliana* (Stockinger et al., 1997), resurrection plant species, *Craterostigma plantagineum* (Mariaux et al., 1998), tomato (Thompson et al., 2000), Bermuda grass, *Cynodon* spp. (Hu et al., 2010), wild emmer wheat (Ergen et al., 2009), and barley (Gürel et al., 2016).

Abscisic acid (ABA) is an important plant hormone produced in response to drought and dehydration. It has long been known that ABA targets stomatal closure as an important rapid response to minimize water loss (Boyle et al., 2016). It was shown in various plant species that ABA is synthesized initially in leaf tissue and then in roots (Ikegami et al., 2009; Goodger and Schachtman, 2010; Seo and Koshiba, 2011), and the gene expression responses of rapidly dehydrated plants or detached leaves are different from those in leaves of intact plants grown under slowly developing drought (Ergen and Budak, 2009; Ergen et al., 2009). During the rapid dehydration of whole plants or detached leaves, in the absence of outside signaling systems, stomatal closure is completely controlled by ABA produced in the leaves and genes responding to dehydration within the leaf signaling system (Ikegami et al., 2009; Goodger and Schachtman, 2010). These are rapid and short-term responses over a few minutes or hours of dehydration. In contrast, during a slower developing drought, where not only leaves but roots and other parts of the plant are involved, stomatal closure is affected by initial rapid ABA production in leaves, followed by the later slower release of ABA as a signal produced from drought-affected roots (Ikegami et al., 2009; Goodger and Schachtman, 2010; Martin-Vertedor and Dodd, 2011; Boyle et al., 2016; Jones, 2016; Verma et al., 2016; Vishwakarma et al., 2017). Whole plants pulled from hydroponics and placed on paper towels go through rapid dehydration that usually results in rapid ABA signaling, which was earlier described as a short-term dehydration treatment over several hours (Ergen et al., 2009; Gürel et al., 2016). Exogenous application of ABA to intact plants and detached leaves can be used to confirm the proposed role of ABA as a signaling agent of drought and dehydration (Boyle et al., 2016).

Transcription factors (TFs) play an important role in the regulation of plant responses to drought and dehydration. Drought Responsive Element Binding (*DREB*) genes have been reported as regulatory components of abiotic stress responses and, particularly, drought response (Zhu, 2002; Agarwal and Jha, 2010; Fujita et al., 2011; Kuromori et al., 2014; Rehman and Mahmood, 2015; Sah et al., 2016; Verma et al., 2016; Agarwal et al., 2017). Recently, we identified that expression levels of the *TaDREB5* gene, which belongs to the *DREB2*-type TFs, correlate with yields of wheat varieties grown in the dry conditions of Kazakhstan (Shavrukov et al., 2016). Application of the Amplifluor-like SNP marker KATU-48, developed in our laboratory, showed a genetic polymorphism associated with two groups of Kazakh wheat genotypes. A significant down-regulation of *TaDREB5* transcript production was observed in dehydrated detached leaves of low-yield wheat varieties compared to non-stressed controls (Shavrukov et al., 2016). However, evaluation of the *TaDREB5* expression levels in leaves of the intact, soil-grown, drought-affected plants of the same wheat genotypes was not assessed.

Nuclear Factor Y (NF-Y), also known as CCAAT Binding Factor (CBF) or Heme Activator Protein (HAP), is a complex TF, which is found in all eukaryotic organisms (Swain et al., 2017; Zanetti et al., 2017; Zhao et al., 2017). In *Arabidopsis* and other plant species, NF-Y TFs play diverse roles in plant development and stress responses, including: flowering time regulation, gametogenesis, embryogenesis, seed development, primary root elongation, response to endoplasmic reticulum stress, hypocotyl elongation, plant performance, ABA signaling, and drought tolerance (Mantovani, 1999; Gusmaroli et al., 2001; Nelson et al., 2007; Petroni et al., 2012; Kuromori et al., 2014; Qu et al., 2015; Yadav et al., 2015; Sah et al., 2016; Swain et al., 2017; Zanetti et al., 2017; Zhao et al., 2017). The majority of NF-Y genes are highly expressed under drought or dehydration (Lee et al., 2015), but some were reported to be down-regulated in response to restricted water availability, such as: *OsNF-YA9* during rapid dehydration of rice plants (Lee et al., 2015), *SiNF-YA5*, *-A7* and *-B2* in simulated drought with polyethylene glycol in foxtail millet plants (Feng et al., 2015), *StNF-YC4* in potato plants subjected to slow drought (van Muijen et al., 2016), and *PmNF-YA3*, *-A4* and *-C3* in leaves of Chinese plum with mannitol simulated dehydration (Yang et al., 2016).

The NF-Y TFs consist of three different subunits: NF-YA, NF-YB, and NF-YC. The presence of all three subunits is essential for binding to the DNA *cis*-element named the CCAAT box (Nardini et al., 2013). In yeast and mammals, each subunit of NF-Y is encoded by a single gene, which may have multiple splicing forms, (Li et al., 1992; Mantovani, 1999), whereas in plants, each NF-Y subunit is encoded by multiple genes (Siefers et al., 2009; Petroni et al., 2012).

The overexpression of the *NF-YC9* gene confers ABA hypersensitivity, resulting in rapid stomatal closure after exposure of *Arabidopsis* seedlings to ABA treatment (Bi C.et al., 2017) due to physical interactions between NF-YC9 and the ABA-responsive bZIP transcription factor ABA-INSENSITIVE5 (ABI5). In rice, several NF-YC subunits were reported to be involved in stress tolerance and plant performance under stress. For instance, overexpression of a rice gene encoding the NF-YC subunit designated Heme Activator Protein gene (*OsHAP2E*) confers resistance to pathogens, tolerance to salinity and drought, and increases photosynthetic rate and tiller number (Alam et al., 2015). Analysis of 70 Ac/Ds rice mutants for salinity tolerance identified one activation-tagged salt tolerant DS plant (DS-16, T3 generation), which showed enhanced expression of a gene encoding the NF-YC subunit, named *OsNF-YC13*. The authors identified it as a possible salt stress tolerance gene (Manimaran et al., 2017). In transgenic rice plants, overexpression of a *NF-YC* gene from Bermuda grass, *CdtNF-YC1*, conferred tolerance to drought and salinity (Chen et al., 2015). However, in potato, the expression of the *StNF-YB4* gene was significantly down-regulated after 4 and 9 days of severe drought stress (van Muijen et al., 2016).

In our previous study, *TaNF-YC15* was identified as the interacting partner of *TaNF-YB2* and *TaNF-YB4* subunits, which play a role in wheat productivity, including grain yield under drought (Nelson et al., 2007; Yadav et al., 2015). The expression of this gene was studied in bread wheat in response to both slowly developing drought and rapid dehydration of detached leaves. Interestingly, the expression of *TaNF-YC15* under these two stresses was different: under drought, levels of *TaNF-YC15* were initially increased and then slowly decreased, while leaf dehydration led to about 3-fold decrease in gene expression (Yadav et al., 2015).

This study aimed: (1) to use an Amplifluor-like SNP marker to reveal an association between the *NF-Y* gene and the yield-under-drought trait; (2) to compare expression profiles of the identified *TaNFYC-A7* gene with the previously reported *TaDREB5* (Shavrukov et al., 2016) in leaves of wheat grown under drought and subjected to rapid dehydration; and (3) to examine stomatal conductance and changes in *TaNFYC-A7* and *TaDREB5* expression following ABA treatment of wheat plants grown in hydroponics. We found that *TaDREB5* expression correlates with grain yield under both drought and dehydration, while correlation of the *TaNFYC-A7* expression with grain yield is dependent on the type of stress. The identified correlations can be potentially used as markers for the selection of cultivars with high yield under drought in the process of conventional breeding.

## Materials and Methods

### Plant Material and Plant Growth

Twelve local wheat varieties representing two groups with contrasting yields were selected from local varieties tested in the field trials, based on their grain yields under the dry weather conditions of Central and Northern Kazakhstan (Shavrukov et al., 2016). Seeds were obtained from the Karaganda Research Institute of Plant Industry and Breeding (Karaganda, Kazakhstan) and pre-germinated in Petri dishes. Twenty-four uniform seedlings of each variety were transplanted into plastic containers (60 × 25 × 25 cm), filled with equal volumes of commercial soil potting mix (Nesterovskoe, Astana, Kazakhstan) and clay soil from a nearby research field, with two containers per variety. Plants were grown for 1 month in controlled environment ‘Phytotron’ chambers at S.Seifullin Kazakh AgroTechnical University, Astana (Kazakhstan), with 24°C/18°C, day/night, light (LED) with photon flux density of 800 μmol m^−2^ s^−1^, and relative humidity of 40%, watered with tap-water three times per week. Placement of containers within growth chambers was fully randomized.

### Experiment 1. Slowly Developing Drought Stress in Whole Plants

For each variety, one container was used for drought treatment and another one for well-watered control. Three 1-month old plants of each variety (three biological replicates) were randomly selected from each container, before drought treatment (designated as Day 0), and the youngest fully developed leaves were collected individually into 10-ml plastic tubes. Leaf samples were immediately frozen in liquid nitrogen and stored at −80°C until RNA extraction. Subsequently, watering was withheld in one of containers for 12 days, while watering was continued in the second container. Volumetric water content (VWC) in the soil was measured using a portable moisture meter (Model CS616, Campbell Scientific, Australia). When VWC value was decreased in the drought treatment approximately two-fold, from 40% field soil capacity to 20% (mild drought), the first symptoms of leaf wilting were observed. At this point, replicate leaves were collected from three independent plants from both drought-treated and well-watered containers and immediately frozen in liquid nitrogen and stored at −80°C until RNA extraction.

### Experiment 2. Rapid Dehydration of Detached Leaves

On the same day (Day 12) of drought treatment, six other plants of each variety were randomly selected from the well-watered controls. For each of the six varieties, six leaves were collected. Three leaves were frozen immediately in liquid nitrogen and stored at −80°C as three biological replicates of controls for further RNA extraction. The other three leaves of each variety were dehydrated on the bench at room temperature (22°C) for 6 h until wilting was clearly observed, as described earlier (Shavrukov et al., 2016), then frozen and stored as above.

### Experiment 3. Rapid Dehydration of Whole Plants and Detached Leaves From Mini-Hydroponics

Four wheat varieties were selected from the 12 used in Experiments 1 and 2. Akmola 2 and Karabalyksyaya 92, identified earlier as genotypes with high-yield potential in the dry environment of Northern and Central Kazakhstan, and Astana 2 and Yugo-Vostochnaya 2, identified as low-yielding (Shavrukov et al., 2016). After 4 days germination, seedlings were transferred to two mini-hydroponic boxes after 1 ml tips, each with a volume of 500 ml (see Figure 5C in Shavrukov et al., 2012). The growth solution was changed every 3 days and it was set up under controlled greenhouse conditions with aeration as published earlier (Shavrukov et al., 2012).

For rapid dehydration experiments, 2-week-old whole plants were sampled from the mini-hydroponics with no obvious root damage. A sample of youngest fully developed leaves was immediately collected, frozen in liquid nitrogen, and designated as ‘Zero-time.’ The remainder of the plants were placed on paper towels for rapid dehydration at constant temperature in the same air-conditioned greenhouse (approximately 22°C). Leaves were similarly sampled from plants after 0.5, 1.5, 6, and 10 h exposure of whole plants to rapid dehydration on paper towels.

In a further experiment with plants from mini-hydroponics, the youngest fully developed leaves were initially detached from the plants and individually dehydrated on paper towels as above with samples being collected at the same time-points. The water loss data for all wheat genotypes used in this paper were reported earlier (Shavrukov et al., 2016).

### ABA Treatment

ABA (+) abscisic acid (Astral Scientific, Australia) was dissolved in ethanol and diluted in growth solution to a final concentration of 100 μM of ABA. For whole plant feeding, roots were immersed in solution, and the youngest fully developed leaves were sampled immediately prior the ABA treatments (‘Zero-time’) and then leaves were progressively sampled at 0.5, 1.5, 6, and 10 h of the treatment for gene expression analyses. Control plants were in growth solution containing ethanol but no ABA. For stomatal conductance experiments, either intact plants were transferred, or individual leaves detached from plants in mini-hydroponics and fed in beakers with growth solution with or without 100 μM ABA.

### Stomatal Conductance

Stomatal conductance (SC), *g_s_* (moles of H_2_O m^−2^ s^−1^) was measured with a LI-COR, Model LI-6800 (LI-COR Biosciences, Lincoln, NE, United States) using a 30 × 20 mm area chamber, with real-time output, as described by the manufacturer. The LED light source was used at 800 μmol m^−2^ s^−1^ PAR. The middle part of the youngest fully developed leaf of intact hydroponic plants was placed in the chamber, and after 30 min of stabilization, SC was measured for 10 min following the experimental treatments. Either whole plants or detached leaves were subjected to dehydration on paper towels or to ABA treatments as above. Data were recorded automatically and retrieved later. Four biological replicates for each genotype and treatment were used in two separate measurements (morning and afternoon) to minimize physiological fluctuations in plant responses.

### DNA Extraction and Amplifluor-Like SNP Analysis

Single leaves, combined from five randomly selected plants from each variety at the tillering stage, were collected in 10-ml plastic tubes and frozen at −80°C prior to DNA extraction. Leaf samples were transferred from liquid nitrogen and ground using two 9-mm stainless ball bearings using Vortex mixer. A phenol-chloroform method of total DNA extraction was used as described earlier (Sharp et al., 1988), and the quality of isolated DNA was checked by PCR.

Amplifluor-like SNP analysis (Myakishev et al., 2001) was carried out using a QuantStudio-7 Real-Time PCR instrument (ThermoFisher Scientific, United States) as described previously (Shavrukov et al., 2016; Jatayev et al., 2017) with following adjustments. Each reaction contained 2xMaster-Mix to give the following final concentrations: 1xPCR Buffer, 2.5 mM MgCl_2_, 0.25 μM of each fluorescent label Universal probe, 0.2 mM each of dNTPs, 0.15 μM of each of two forward primers, 0.78 μM of common reverse primer and 0.02 units of Taq DNA polymerase (Maxima, ThermoFisher, United States). Five μl of Low ROX (ThermoFisher, United States) was added as a passive Reference label to the entire Master-mix volume as prescribed for the qPCR instrument prior loading of 5 μl Master-mix in 96-well microplate. Another half of the PCR volume (5 μl) in each well was genomic DNA, adjusted to 10 ng/μl. Sequences of the used Universal probes and primers as well as sizes of amplicons are present in **Supplementary Material 1**.

PCR was conducted using a program adjusted from those published earlier (Rickert et al., 2004; Khripin, 2006): initial denaturation, 95°C, 1 min; 20 ‘doubled’ cycles of 95°C for 10 s, 55°C for 10 s, 72°C for 20 s, 95°C for 10 s, 50°C for 20 s, and 72°C for 50 s; and final extension for 72°C for 1 min (Jatayev et al., 2017). Genotyping with SNP calling was determined automatically by instrument software, but each SNP result was also checked manually using amplification curves and final allele discrimination. Experiments were repeated twice over different days, where technical replicates confirmed the confidence of SNP calls.

### RNA Extraction, cDNA Construction and qPCR Analysis

Frozen leaf samples were ground as described above for DNA extraction. TRIsol-like reagent was used for RNA extraction following the protocol described by Shavrukov et al. (2013) and RNA quality checked on agarose gels. After DNase treatment with 1 μl of DNase per reaction (Qiagen, Germany), the cDNA was constructed using a MoMLV Reverse Transcriptase kit (Biolabmix, Novosibirsk, Russia) with 2 μg of each RNA sample, oligo(dT)_20_ primer and dNTPs as recommended by the manufacturer. All cDNA samples were checked for quality using PCR and yielded bands of the expected size on agarose gels.

Diluted (1:2) cDNA samples were used for qPCR analyses using both a QuantStudio-7 Real-Time PCR instrument (ThermoFisher Scientific, United States) at Kazakh AgroTechnical University, Astana, Kazakhstan, and Real-Time qPCR system, Model CFX96 (BioRad, Gladesville, NSW, Australia) at Flinders University, Australia. The qPCR protocol was similar in both instruments as published earlier (Shavrukov et al., 2016), wherein the total volume of 10 μl qPCR reactions included 5 μl of 2xKAPA SYBR FAST (KAPA Biosystems, United States), 4 μl of diluted cDNA, and 1 μl of mixed two gene-specific primers (3 μM of each primer) (**Supplementary Material 1**). Expression data for the target genes were normalized using the average expression values of two reference genes: Ta2291, ADP-ribosylation factor (*ADPRF*) and Ta30768, Glyceraldehyde-3-phosphate dehydrogenase (*GAPDH)* (Paolacci et al., 2009). At least three biological and two technical replicates were used in each qPCR experiment.

### Statistical Analysis

Means and standard errors were calculated with ANOVA using Windows Excel software; and probabilities for significance were estimated using Student’s *t*-test.

## Results

### Genotyping of Wheat Varieties Using Amplifluor-Like SNP Markers for *TaNFYC-A7* and *TaDREB5*

**FIGURE 1:**
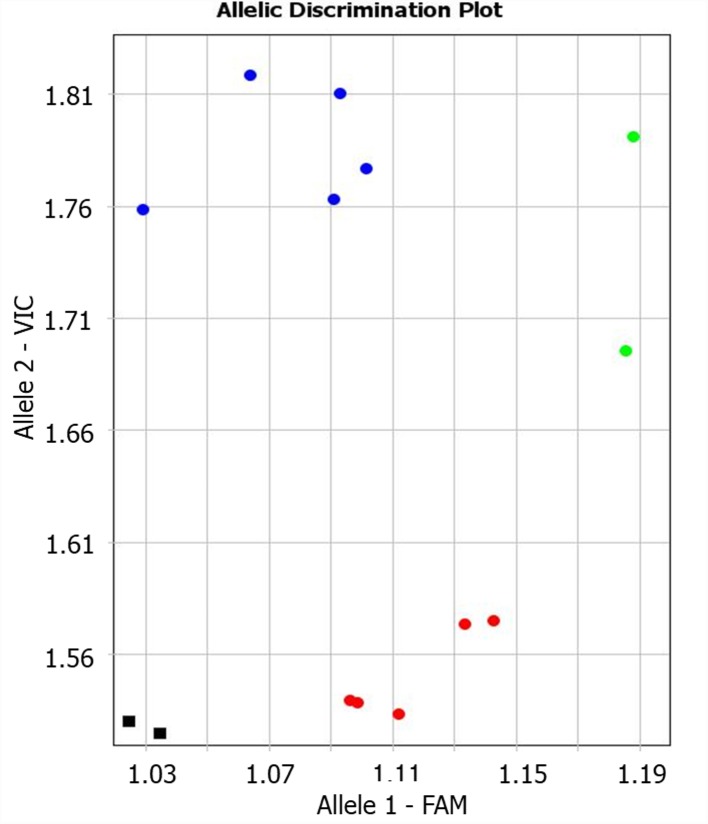
Results of allelic discrimination of Amplifluor-like SNP marker KATU-W58 in twelve selected bread wheat varieties from Kazakhstan. Red and blue dots indicate automatic SNP calls for homozygotes in Allele 1-FAM (*aa*), and Allele 2-VIC (*bb*), associated with high- and low-yielding wheat varieties, respectively. Green dots indicate heterozygotes (*ab*) or mixed genotypes. Black squares show NTC (No Template Control).

The candidate gene *TaNFYC-A7* was identified among other NF-YC genes as containing polymorphic SNP suitable for analysis of wheat varieties, and the Amplifluor-like SNP marker, KATU-W58, was developed for *TaNFYC-A7* genotyping (**Supplementary Material 2**). A clear distinction between the two groups of selected wheat varieties was apparent using the KATU-W58 marker (**Figure 1**). Five varieties from the high-yield group had Allele 1 (labeled with FAM), while Allele 2 (labeled with VIC) was found in five varieties from the low-yield group. Genotypes of two varieties (Saratovskaya 55 and Saratovskaya 60) were determined as heterozygous or mixed.

The previously identified SNP marker KATU-48 for the *TaDREB5* gene was used in this study and genotyping was repeated (data not shown). The results obtained were very similar and the genotyping score identical, confirmatory of those published earlier (Shavrukov et al., 2016).

### Experiments 1 and 2. *TaDREB5* and *TaNFYC-A7* Expression in Drought-Stressed Plants and in Dehydrated Detached Leaves

**FIGURE 2:**
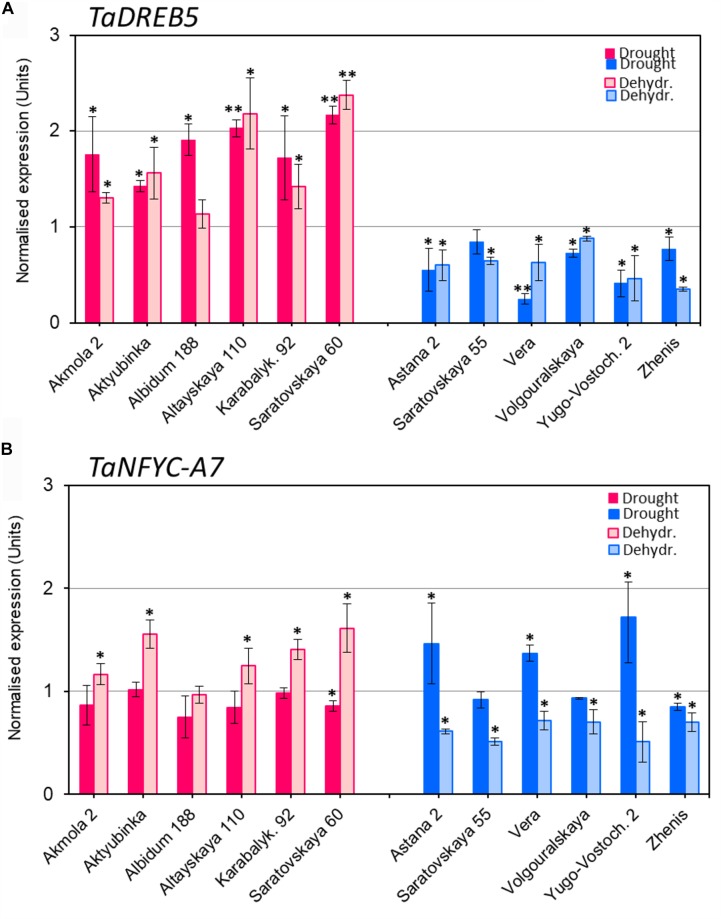
Expression of *TaDREB5* **(A)** and *TaNFYC-A7* **(B)** in whole plants under drought and in dehydrated detached leaves. Six high-yielding (in red/pink, left side of images **A,B**) and six low-yielding wheat varieties (in blue, right side of images **A,B**) were selected based on yield data obtained in the field using wheat varieties grown in the dry conditions of Central and Northern Kazakhstan. *Drought:* One-month old plants grown in soil in controlled greenhouse conditions were subjected to slowly developing drought by withdrawal of watering for 12 days. *Dehydr.:* Detached leaves from well-watered plants were subjected to 6 h dehydration at room temperature. Expression data for genes *TaDREB5* and *TaNFYC-A7* represent Means ± SE for three biological replicates and two technical replicates in qPCR experiments, calculated with ANOVA, where significant differences are indicated (^∗^*P* > 0.95; and ^∗∗^*P* > 0.99) compared to corresponding controls, according to Student’s *t*-test. Expression levels in leaves of control (non-treated) wheat plants for each variety were set to one unit. Data were normalized using the average expression levels of two reference genes. More details are available in **Supplementary Material 1**.

Expression of *TaDREB5* showed clear differences between the two groups of wheat varieties after both drought and dehydration treatments (**Figure 2A**). Under drought stress, *TaDREB5* transcripts were 1.5- to 2.1-fold higher than in control plants. Similarly, increased expression was observed in five of the six high-yielding varieties subjected to rapid dehydration, while the sixth, Albidum 188, had the same level of *TaDREB5* mRNA as the control (**Figure 2A**, red/pink columns). In contrast, *TaDREB5* expression was decreased to 0.2- to 0.9-fold in the low-yield group after both drought and dehydration treatments. The changes in expression levels were small but mostly significant in all six low-yielding varieties (**Figure 2A**, blue column).

The expression patterns of *TaNFYC-A7* in wheat were different under slowly developing drought or rapid dehydration treatments (**Figure 2B**). Most high-yielding varieties showed no change in *TaNFYC-A7* expression level under drought conditions. Low-yielding varieties comprised two groups: Astana 2, Vera and Yugo-Vostochnaya 2 had significantly higher (1.4–1.7-fold) expression of *TaNFYC-A7* under drought, while Saratovskaya 55, Volgouralskaya and Zhenis showed no difference or slightly decreased expression levels (**Figure 2B**, dark blue column).

In contrast, the expression of *TaNFYC-A7* was significantly increased in dehydrated leaves of most of the high-yielding group (**Figure 2B**, pink column). All of the low-yielding group showed significant decreases in *TaNFYC-A7* expression after dehydration treatment (**Figure 2B**, light blue column).

### Experiment 3. *TaDREB5* and *TaNFYC-A7* Expression in Rapidly Dehydrated Intact Plants, Detached Leaves, and in Response to ABA Treatment

**FIGURE 3:**
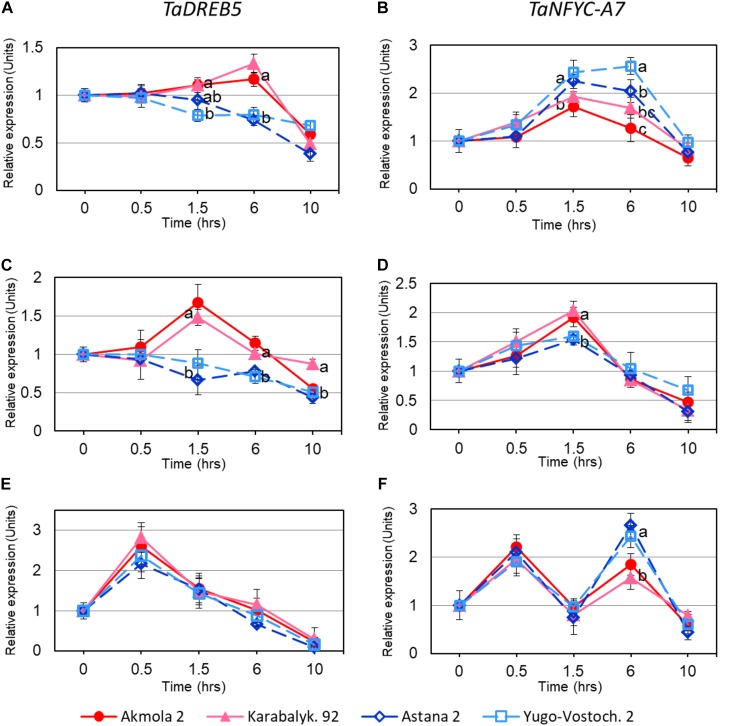
Expression levels of *TaDREB5* and *TaNFYC-A7* in rapidly dehydrated intact plants **(A,B)**, detached leaves **(C,D)**, and in intact plants after ABA treatment **(E,F)**. Names of the four studied varieties are shown in the bottom of the Figure. Two week-old plants grown in mini-hydroponics in controlled greenhouse conditions were pooled, and whole plants as well as detached leaves were subjected to rapid dehydration at room temperature. Expression data for genes *TaDREB5* and *TaNFYC-A7*, represent Means ± SE for three biological replicates and two technical replicates of qPCR experiments, calculated with ANOVA, where significant differences (*P* > 0.95) according to Student’s *t*-test, are indicated by different letters in the same time-points. Expression levels in leaves of control (non-treated) plants for each variety were set to one unit. Data were normalized using the average expression of two reference genes.

Both *TaDREB5* and *TaNFYC-A7*, showed transcriptional responses after 1.5 h of whole plant dehydration. High- and low-yielding wheat varieties displayed different expression profiles: Akmola 2 and Karabalykskaya 92 (high-yield, red and pink in **Figure 3**) recorded increased expression for both genes at 1.5 and 6 h, and then returned back to the initial level or below after 10 h (**Figures 3A,B**). In low-yield varieties (Astana 2 and Yugo-Vostochnaya 2; dark and light blue in **Figure 3**), expression of *TaDREB5* and *TaNFYC-A7* significantly either decreased, or increased, respectively, at the same time-points (**Figures 3A,B**).

In dehydrated detached leaves, the trend in *TaDREB5* expression was similar to that in dehydrated intact plants, but elevated expression in high yield varieties started earlier, and reached maximum at 1.5 h (**Figure 3C**). The pattern of *TaNFYC-A7* expression with dehydration was somewhat different in detached leaves compared to that in whole plants, being significantly higher at 1.5 h of dehydration in the high-yielding varieties (**Figure 3D**).

During the treatment of intact plants with ABA, high levels of transcript production from both genes were found during the first 30 min, which subsequently decreased to the initial level after 1.5 h treatment. Following these rapid responses, *TaDREB5* expression further declined at 6 and 10 h of ABA treatment for all four wheat genotypes (**Figure 3E**). However, for the *TaNFYC-A7* gene, a different pattern comprising a second peak of strong expression was observed at 6 h of ABA treatment (**Figure 3F**). Expression again decreased 10 h after ABA application. This unexpected, double-peak profile of *TaNFYC-A7* expression, clearly unlike the single peak of *TaDREB5*, may indicate that these two genes are regulated differently.

### Stomatal Conductance

**FIGURE 4:**
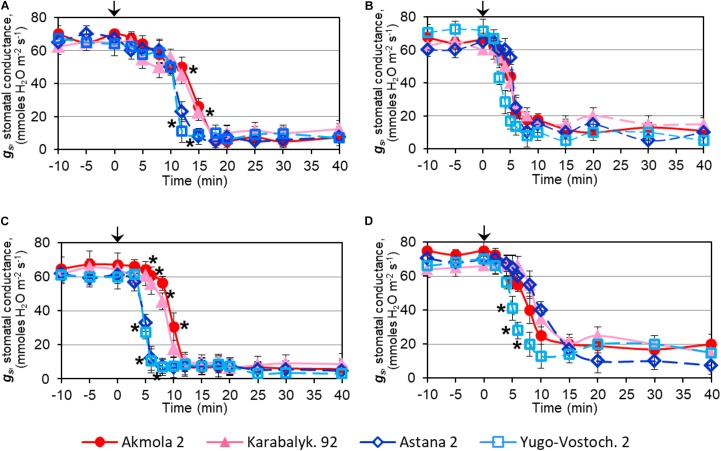
Stomatal conductance, *g_s_*, in leaves of wheat plants during: dehydration of intact plants **(A)**, detached leaves **(B)**, after treatment with 100 μM ABA on intact plants **(C)**, and detached leaves **(D)**. Names of the four studied varieties are shown in the bottom of the Figure. Leaves of 2-week-old plants grown in mini-hydroponics in controlled conditions of the greenhouse were placed in the measuring chamber with inbuilt light source. Whole plants and detached leaves were subjected either to quick dehydration on paper towels at room temperature or to treatments with 100 μM ABA. The starting points of the treatment (‘Zero-time’) are indicated by arrows at the top of each Figure panel. Data are shown as Means ± SE (*n* = 4), calculated with ANOVA, where significant differences (*P* > 0.95) according to Student’s *t*-test, are indicated by (^∗^) in the same time-points.

Rapid dehydration of whole plants resulted in a strong decrease in SC due to stomatal closure. The reduction of SC was initially detected after 5 min of dehydration and dropped sharply between 10 and 15 min. The two high-yielding varieties showed a significant delay in SC reduction compared to low-yielding wheat varieties (**Figure 4A**). In detached leaves, the major decline of SC occurred about 5 min after the start of dehydration, earlier than in intact plants, and no significant differences were observed among the wheat varieties (**Figure 4B**).

In contrast to dehydration, ABA treatment resulted in a more rapid effect with a decline of SC within 5–10 min of treatment. However, the high-yielding varieties showed a significant delay in SC reduction of about 5 min compared to low-yielding varieties (**Figure 4C**). Similarly, a lowering of SC was observed between 5 and 10 min of ABA treatment in detached leaves, but only in one low-yielding variety, Yugo-Vostochnaya 2, was a significantly earlier reduction of SC evident (**Figure 4D**).

## Discussion

The physiological responses of plants to slowly developing drought or rapid dehydration of either whole plants or detached leaves have some important differences. In the first case, there is a coordinated systemic response involving the entire plant at physiological and molecular levels in response to gradually decreasing water availability and increasing stress. In the second case, in rapidly dehydrated plants or leaves, the response can be attributed mainly to rapid protective changes within the leaves rather than the whole plant. This involves rapid signaling from other plant organs (Ikegami et al., 2009; Goodger and Schachtman, 2010; Waadt et al., 2014; Gürel et al., 2016). For wild emmer wheat and barley, global transcriptome and gene expression analyses have been compared after growth either in greenhouse conditions under slowly developing drought, or subjected to rapid shock-like dehydration of whole plants. It was found that several TFs showed different expression profiles under the two types of stresses in genotypes tolerant or sensitive to drought (Ergen and Budak, 2009; Ergen et al., 2009; Gürel et al., 2016). The present study centered on two genes, *TaDREB5* and *TaNFYC-A7*, to examine whether they may be differentially expressed. Associations between these two genes and the yield-under-drought trait were found using Amplifluor-like SNP markers (Shavrukov et al., 2016). These genes were selected due to the regulatory nature of TFs, which makes them more attractive candidates compared to other genes since they are potentially capable of regulating whole groups of downstream genes responsible for the same trait.

Our earlier work showed differences in expression patterns of *TaDREB5* in wheat varieties from Kazakhstan (Shavrukov et al., 2016) where expression was strongly decreased in all six low-yielding varieties, while it was unchanged or slightly increased in six high-yielding wheat varieties. There were also differences in response to slowly developing drought or rapid dehydration of detached leaves of bread wheat. The present study confirmed these results in the same sets of wheat varieties, including rapid dehydration of hydroponically grown intact plants (**Figures 3A,C**). A more rapid response of *TaDREB5* to dehydration in detached leaves (1.5 h) compared to whole plants (6 h) was evident, probably due to the faster dehydration of detached leaves.

Both high- and low-yielding groups of wheat genotypes showed statistically significant differences in *TaDREB5* gene expression in all three experiments compared to controls (**Figures 2**, **3A,C**). However, no substantial differences were found between slowly drought-affected and rapidly dehydrated plants within each of the two groups of wheat varieties. We can speculate that any signaling system activated by drought or dehydration in whole plants affects transcription of *TaDREB5* very similarly regardless of the slow or rapid application of stress. Dehydrin genes could be amongst the downstream genes targeted by TFs, like *TaDREB5*, during drought and dehydration. Immunoblot analysis of dehydrin polypeptides showed similar activity levels both in whole drought-stressed plants and in dehydrated detached leaves of Bermuda grass, *Cynodon* spp. (Hu et al., 2010), similar to the expression pattern of *TaDREB5* pattern in our experiment. Expression profiles of other *DREB* genes in wheat were reported in a number of papers, e.g., for *TaDREB1* in the Chinese wheat cultivar Xiaoyan 54 (Shen et al., 2003) and for *TaDREB2* and *TaDREB3* in the Australian drought tolerant wheat variety RAC875 (Morran et al., 2011). *DREB* gene expression levels in wheat and other tested plants are usually low or very low. They increase several fold when plants are exposed to dehydration and drought (Agarwal et al., 2017).

ABA is a well-documented mediator of signaling in drought-stressed or rapidly dehydrated plants, where the expression of ABA-dependent genes can be affected (Zhu, 2002; Agarwal and Jha, 2010; Fujita et al., 2011; Jones, 2016). Comparison of ABA-dependent and ABA-independent gene responses in plants can therefore provide useful information about the regulation of stress-related genes, including regulatory genes. This study complements those reported earlier on the ABA-biosynthetic genes, zeaxanthin epoxidase (ZEP) and 9-*cis*-epoxycarotenoid dioxygenase (NCED). These genes are differentially expressed in response to slowly developing drought in plants and rapidly dehydrated leaves of tomato (Thompson et al., 2000).

The *TaNFYC-A7* gene identified with SNP marker in this work is one of the *TaNF-YC7* homeologues located in the A genome of bread wheat. A phylogenetic tree of NF-YC proteins containing TaNFYC-A7 under the name TaNF-YC7 can be found in Figure 2B in Yadav et al. (2015). It is obvious that TaNFYC-A7 belongs to the same clade as TaNF-YC15 described in this paper. TaNF-YC15 was found in the Y2H screen with ZmNF-YB2a, which was previously reported to be responsible for increased yield under drought in transgenic maize (Nelson et al., 2007). TaNF-YC15 was also isolated in another Y2H screen using the TaNF-YB4 subunit as bait. Constitutive TaNF-YB4 overexpression in wheat led to a significant increase in grain yield under well-watered conditions (Yadav et al., 2015). These findings lead us to speculate that TaNFYC-A7, as a close homologue of TaNF-YC15, may play a role in the regulation of plant productivity.

Both high and low yielding wheat groups showed variable expression of *TaNFYC-A7* in both drought-stressed and rapidly dehydrated plants. There are similar reports elsewhere in the literature that show in some cases that the majority of *NF-Y* genes were highly expressed under drought or dehydration (Lee et al., 2015; Yang et al., 2016), compared to others that recorded a decrease (Feng et al., 2015; Lee et al., 2015; van Muijen et al., 2016; Yang et al., 2016). Three wheat genes, *TaNF-YC5*, *TaNF-YC11* and *TaNF-YC12*, were reported to be down-regulated by drought (Stephenson et al., 2007). *TaNF-YC15* was initially up-regulated and later down-regulated by both drought and dehydration (Yadav et al., 2015). Expression profiles for *TaNF-YC7* to our knowledge have not yet been reported, and in the current study, changes in the expression of this gene in different wheat varieties in response to drought and dehydration are presented for the first time.

High-yielding varieties showed no change or minor reduction in *TaNFYC-A7* expression in leaves of slowly drought-stressed plants, but expression was significantly increased in rapidly dehydrated leaves of most wheat genotypes (**Figure 2B**, pink columns). Plants from the low-yielding group showed variation in expression levels of *TaNFYC-A7*, but it was generally higher in leaves after rapid dehydration compared to slowly drought-stressed plants in all six genotypes (**Figure 2B**, blue columns). These results were also confirmed in Experiment 3.

These findings indicate that *TaNFYC-A7* expression was possibly dependent on the presence or absence of signaling systems operating in the intact wheat plants under drought but not in rapidly dehydrated whole plants or detached leaves. In *Arabidopsis*, it has been shown that genes *NF-YC3*, *-C4* and *-C9* act together in a single hub regulated by ABA signaling (Warpeha et al., 2007; Kumimoto et al., 2013). We decided to test our hypothesis that the differences in response between slowly developing drought and rapid dehydration in wheat may be determined by the ABA signaling cascade. Due to technical feasibility, ABA-dependent expression of *TaNFYC-A7* and *TaDREB5* were compared using a hydroponic system. For both genes, expression was strongly evident after only 30 min of ABA application, while a second, later peak for *TaNFYC-A7* expression suggested that only this gene is strongly regulated by ABA (**Figures 3E,F**). Previously, single peaks have been reported as significantly increased for various NF-Y TFs (Lee et al., 2015; Yang et al., 2016; Bi C.et al., 2017; Na et al., 2017; Sun et al., 2017). Only a few examples, such as *PmNF-YB6* and *PmNF-YC5*, show significant double-peak up-regulation, measured in young leaves of Chinese plum after 3, 6, and 24 h of ABA treatment (Yang et al., 2016). Therefore, our results reveal an unusual pattern of *TaNFYC-A7* expression with two peaks of up-regulation in wheat.

Stomatal closure, a classic initial response in plants evolved to minimize water loss under drought and dehydration, is strongly regulated by the ABA signaling system (Farquhar and Sharkey, 1982; Ikegami et al., 2009; Goodger and Schachtman, 2010; Seo and Koshiba, 2011; Boyle et al., 2016; Jones, 2016). Measurement of stomatal conductance, SC, can indicate how fast plants can react to the stress, and there are several reports concerning the regulation of SC and stomatal closure by TFs. Overexpression of *TaNF-YB3;1* in tobacco and *StNF-YB3.1* in potato were reported to cause stomatal closure, enhancing the expression of several ABA-related genes (Xuanyuan et al., 2017; Yang et al., 2017), while overexpression of *StNF-YA7*, encoding a different subunit (NFY-A), reduced water loss and improved tolerance to slow drought in transgenic potato compared to controls (Na et al., 2017). When intact non-stressed plants were treated with ABA in the present studies, they reacted sooner, due to the presence of an intact root system, with SC declining within 5–10 min while detached leaves required 5–15 min (**Figures 4C,D**). Compared to the high-yielding varieties, SC dropped significantly earlier for some of the low-yielding wheat varieties by about 5 min in two whole plant genotypes and in detached leaves of one genotype, respectively (**Figures 4C,D**). We can see from these experiments that stomatal closure is strongly regulated by ABA, similar to earlier reports (Reviewed by Seo and Koshiba, 2011; Jones, 2016). For example, the content of endogenous ABA was significantly increased in leaves of drought stressed *Arabidopsis* (Ikegami et al., 2009) and maize (Goodger and Schachtman, 2010) plants, and this was accompanied by stomatal closure. In addition, it was reported that the increase in endogenous ABA after water withholding or application of exogenous ABA had the same effect on stomatal closure in *Pelargonium* (Boyle et al., 2016).

Dehydration of whole plants or detached leaves showed similar results, with rapid closure of stomata after approximately 10 or 5 min, respectively, from the start of ABA application (**Figures 4A,B**). However, it was found that high-yielding wheat genotypes showed a significant delay of about 5 min in decrease of SC compared to the low-yielding varieties. These results are in accordance with those described above for potato, with overexpression of *StNF-YB3.1* causing stomatal closure which also was associated with a significant reduction in tuber yield (Xuanyuan et al., 2017). In bread wheat in water-limited conditions, significant positive correlations between stomatal characteristics (width, length and area) and grain yield were reported (Arminian et al., 2008). Our results suggest that wheat plants which close their stomata more slowly are associated with better yield performance in the field. This interpretation is consistent with the observation that wheat cultivar Drysdale showed higher ABA accumulation in leaves and quicker stomatal closure compared to the superior drought-tolerant breeding line IGW-3262 when water was withheld (Saradadevi et al., 2014, 2016). It appears that plants can coordinate TF gene expression and ABA production to regulate down-stream genes affecting stomatal closure; in turn, this influences tolerance to drought and rapid dehydration, as well as final biomass and seed production (Flexas and Medrano, 2002). In addition, it is important that water loss from wheat leaves depends not only on the size, density and behavior of stomata, but can be also regulated by the thickness and/or molecular content of the leaf cuticle (Jäger et al., 2014; Bi H. et al., 2017; Bi et al., 2018). The water loss of wheat varieties examined in this work was found to correlate well with grain yield under drought (Shavrukov et al., 2016).

## Conclusion

A clear correlation exists between the expression levels of two TFs, *TaDREB5* and *TaNFYC-A7*, in leaves of plants exposed to slowly developing drought or rapid dehydration of either intact plants or detached leaves, and grain yields of wheat varieties grown in the field under water limiting conditions (**Figures 2**, **3**). Expression levels of *TaDREB5* were significantly increased in high-yielding varieties compared to controls regardless of which kind of stress was applied. In contrast, *TaNFYC-A7* expression profiles were more complex. Under slowly developing drought, high- and low-yielding wheat genotypes showed decreased and increased *TaNFYC-A7* expression, respectively. Interestingly, under rapid dehydration of detached leaves, the opposite trend was observed: an increase in *TaNFYC-A7* expression levels in nearly all high-yielding varieties tested, but a reduction in all low-yielding genotypes. These findings need to be further elaborated in future studies using a wider range of wheat varieties with contrasting yield-under-drought characteristics. If confirmed, changes in the expression of *TaDREB5* and the ratio of *TaNFYC-A7* expression under drought and dehydration treatments could potentially become useful molecular markers in breeding for the prediction and selection of high-yield genotypes for yield-under-dry conditions. Generation of expression data was just the first step in the analysis of gene function; further characterisation of the candidate genes described in this paper, and other genes identified in our project related to yield-under-drought, is ongoing.

## Author Contributions

LZ conducted the genotyping Amplifluor-like SNP analysis, AK and SJ supervised the experiments and interpreted the results. GK and AZ conducted the experiments with plant stresses and sampling. DS carried out qRT-PCR experiments and prepared the data. SS and TS worked with plants in the field trial, VS coordinated the experiments in the field and sampling. SL analyzed cloned gene sequences and wrote the corresponding section. CJ coordinated experiments with ABA and stomatal conductance and revised the corresponding section. KS coordinated the qRT-PCR study and revised the corresponding section. PL supervised the project, revised and approved the final version of the manuscript. YS coordinated all experiments and wrote the first version of the manuscript.

## Conflict of Interest Statement

The authors declare that the research was conducted in the absence of any commercial or financial relationships that could be construed as a potential conflict of interest.
