# Supplementary material for: Genes Encoding Transcription Factors TaDREB5 and TaNFYC-A7 Are Differentially Expressed in Leaves of Bread Wheat in Response to Drought, Dehydration and ABA
Source: Front Plant Sci. 2018 Sep 27;9:1441. doi: 10.3389/fpls.2018.01441 (PMC6171087; doi:10.3389/fpls.2018.01441)
Supplement: Supplementary file 1 [file Data_Sheet_1.PDF]

Supplementary materials

Supplementary materials 1.

Sequences, targets, sizes of amplicons and references for the Universal probes and primers used. Common fluorescence-specific tails are indicated in Italics. Fluorophores and quenchers are in Bold.

Name	Sequence (5’ – 3’)	Target	Reference
Uni-Probe-1	**FAM**-AGCGATGCGTTCGAGCATCGC**-(T*-BHQ1)**-*GAAGGTGACCAAGTTCATGCT*	Amplifluor-like PCR	Myakishev et al., 2001; Rickert et al., 2004; Khripin, 2006; Jatayev et al., 2017
Uni-Probe-2	**VIC-**AGGACGCTGAGATGCGTCC**-(T*-BHQ1)**-*GAAGGTCGGAGTCAACGGATT*		
KATU-48-F1	*GAAGGTGACCAAGTTCATGCT*GTTGCTTCTTCCTTGCCGA	Amplifluor-like PCR, 75 bp	Shavrukov et al., 2016
KATU-48-F2	*GAAGGTCGGAGTCAACGGATT*GTTGCTTCTTCCTTGCCGC		
KATU-48-R	TCTGTGGACTCAAGCGCATC		
KATU-W58-F1	*GAAGGTGACCAAGTTCATGCT*CCTAATGGTAGCTTCGAC	Amplifluor-like PCR, 99 bp	Current study
KATU-W58-F2	*GAAGGTCGGAGTCAACGGATT*TCCTAATGGTAGCTTCGAT		
KATU-W58-R	AAGGTACTGTTTACTAGATCAG		
KATU-48-Fq	TGTTGCTTCTTCCTTGCCG	qPCR, 75 bp	Current study
KATU-48-Rq	TCTGTGGACTCAAGCGCAT		
KATU-W58-Fq	TTCCTAATGGTAGCTTCGA	qPCR, 97 bp	Current study
KATU-W58-Rq	GGTACTGTTTACTAGATCAG		
ADPRF-F	GCTCTCCAACAACATTGCCAAC	Reference gene, qPCR, 165 bp	Paolacci et al., 2009
ADPRF-R	GCTTCTGCCTGTCACATACGC		
GAPDH-F	TTGCTCTGAACGACCATTTC	Reference gene, qPCR, 175 bp	
GAPDH-R	GACACCATCCACATTTATTCTTC		

Supplementary materials 2.

SNP position and design of allele-specific primers for the Amplifluor-like SNP marker KATU-W58 in wheat. The sequence was retrieved from the Cereal DB database (http://www.cerealsdb.uk.net/cerealgenomics/CerealsDB), Contig BC000018972. The SNP position in the sequence is indicated with a 'Y' highlighted in red, and consists of either a 'C' or 'T' nucleotide. Two forward primers and one common reverse primer are shown in Bold and highlighted in blue and purple, respectively. Amplicon size is indicated. The tails are shown in normal case.

Sequence:

5'-T**TCCTAATGGTAGCTTCGAY**GCTGGTATGGCACCAGCGGTAGGNNNNNNNGATTGTGATTTGT
TAGGTGATTTGAGA**CTGATCTAGTAAACAGTACCTT**CGTTTTCGCCCTAGTTTCTATCTAATC-3'

PCR product size: 99 bp

Primers:

KATU-W58-F1: 5'-GAAGGTGACCAAGTTCATGCT**CCTAATGGTAGCTTCGAC**-3'

KATU-W58-F2: 5'-GAAGGTCGGAGTCAACGGATT**TCCTAATGGTAGCTTCGAT**-3'

KATU-W58-R: 5'-**AAGGTACTGTTTACTAGATCAG**
